# The Overexpression of *SlPLATZ17* Can Increase the Tolerance of Tomatoes to Drought and Salt Stress

**DOI:** 10.3390/ijms26051836

**Published:** 2025-02-21

**Authors:** Xueli Jia, Xinyue Pang, Min Xu, Congmin Wang, Huixin Wei, Jing Liu, He Zhang, Dalong Li, Xiangyang Xu, Tingting Zhao

**Affiliations:** 1 Tomato Research Institute, College of Horticulture and Landscape Architecture, Northeast Agricultural University, Harbin 150030, China; jiaxueli2023@163.com (X.J.); pxinyue1201@163.com (X.P.); 15024995257@163.com (M.X.); 15954153922@163.com (C.W.); weihuixin011014@163.com (H.W.); 18334804395@163.com (J.L.); esculentum@neau.edu.cn (H.Z.); lidalong@neau.edu.cn (D.L.); 2 Key Laboratory of Biology and Genetic Improvement of Horticultural Crops (Northeast Region), Ministry of Agriculture and Rural Affffairs, Northeast Agricultural University, Harbin 150030, China

**Keywords:** tomato, *SlPLATZ17*, gene editing, drought and salt stresses, protein interactions

## Abstract

PLATZ, a novel zinc finger DNA-binding protein, plays crucial regulatory roles in the growth and development of various plants and in the modulation of abiotic stress responses. However, knowledge of the functions of PLATZ genes in tomato (*Solanum lycopersicum*) is lacking. In our study, we established stable *SlPLATZ17* gene overexpression and knockout lines to further analyze the regulatory functions of *SlPLATZ17* under drought and salt stress. It was found that the overexpression lines presented increased drought and salt tolerance under stress. Transcriptome sequencing and screening for interacting proteins revealed that SlPLATZ17 may exert its effects by interacting with POR1, thereby influencing glutathione metabolism and arginine and proline metabolism. In conclusion, the findings lay the groundwork for a deeper understanding of the regulatory mechanisms underlying the role of *SlPLATZ17* in stress resistance, as well as facilitating the application of *SlPLATZ17* in the breeding of stress-resistant tomato varieties.

## 1. Introduction

The growth and development of tomatoes (*Solanum lycopersicum*) are significantly influenced by environmental factors such as light, air, water, and temperature. When tomato cells are subjected to abiotic stress, the stability of the cell membrane is compromised, leading to alterations in membrane protein structure and increased permeability [1]. Abiotic stress can lead to the excessive accumulation of proline in plants, resulting in the production of reactive oxygen species (ROS), which are byproducts of physiological metabolism [2,3]. This process is precisely regulated by both enzymatic and nonenzymatic antioxidant defence systems [4,5,6]. Superoxide dismutase (SOD) disproportionates superoxide anions (O_2_^−•^) into hydrogen peroxide (H_2_O_2_) and O_2_ [7]. Peroxidase (POD) plays distinct roles during various stages of ageing and stress, influencing processes both before and after these events [8]. The physiological changes induced by stress can significantly impact tomato quality and yield. Therefore, it is crucial to investigate the stress resistance mechanisms in tomatoes, explore genetic resources for stress tolerance, and cultivate and select varieties that are resilient to adverse conditions.

PLATZ is a novel type of zinc finger DNA-binding protein. The nomenclature of zinc finger transcription factors is derived from their distinctive finger-like structure and their ability to form specific interactions with zinc ions [9]. It can activate or inhibit gene transcription through three distinct mechanisms [10]. Zinc finger proteins are classified into nine major categories. Zinc finger proteins play crucial roles in the expression and regulation of plant genes, as well as in growth and development, antibacterial immunity, and responses to both abiotic and biotic stresses [11,12,13]. PLATZ1 possesses two distinct structural domains: C-x2-H-x11-C-x2-C-x(4-5)-C-x2-C-x(3-7)-H-x2-H and C-x2-C-x(10-11)-C-x3-C [14].

Numerous reports have confirmed that PLATZ transcription factors play a significant role in plant growth and development and are involved in the response to various abiotic stresses [15,16]. In rice, the *SG6* gene encodes a PLATZ protein. The overexpression of the *G6* gene results in a significant increase in grain size and weight, along with a notable height increase in the plants. This observation is consistent with the high rate of spikelet hull cell division associated with *SG6* [17]. Transgenic Arabidopsis and soybean hairy roots overexpressing *GmPLATZ17* exhibited drought sensitivity [18]. *PLATZ4* interacts with *AITR6* to increase ABA sensitivity and drought tolerance in Arabidopsis by upregulating the expression of PR1, ABI3, ABI4, and ABI5 and suppressing the expression of PIP2;8 and related genes [19]. In vitro and in vivo experiment have shown that in *A. thaliana*, *AtPLATZ2* directly binds to A/T-rich sequences in the CBL4/SOS3 and CBL10/SCaBP8 promoters and represses the activity of the CBL4/SOS3 promoter in the leaves of the plant, thereby inhibiting the salt tolerance of the plant [20]. However, few stress-related studies on these PLATZ family genes have been conducted on tomatoes, and the molecular mechanisms by which tomato PLATZ genes regulate abiotic stress and plant growth and development are unknown.

In a previous study, we examined the *SlPLATZ17* gene, which exhibited a significant response under various stress conditions. Using virus-induced gene silencing (VIGS), we initially demonstrated that *SlPLATZ17* is involved in regulating the tomato plant’s resistance to drought and salt stress. [21]. However, given the limitations of VIGS technology, the function of this gene needs to be further validated. Based on preliminary research findings and the investigation of the PLATZ gene’s role in stress resistance across various species, we can hypothesize that *SlPLATA17* positively regulates the drought and salt tolerance of tomatoes.

In this study, we constructed a stable T2 overexpression strain and a double allelic mutant T2 gene-edited strain of the *SlPLATZ17* gene through genetic transformation, analyzed its response to drought and salt stress, screened for intercalating proteins via yeast two-hybrid technology, and analyzed the metabolic pathways involved in *SlPLATZ17* and its possible involvement in functional modules by combining the transcriptomic data of different strains. We also analyzed the metabolic pathways in which *SlPLATZ17* and its interacting proteins are involved and explored the functional modules in which *SlPLATZ17* might be involved. The key mechanism by which *SlPLATZ17* regulates tomato resistance was ultimately elucidated, laying the foundation for the breeding of resistant tomato plants.

## 2. Results

### 2.1. Expression Pattern of SlPLATZ17 in Different Tissues of Tomato and Its Subcellular Localization

**Figure 1:**
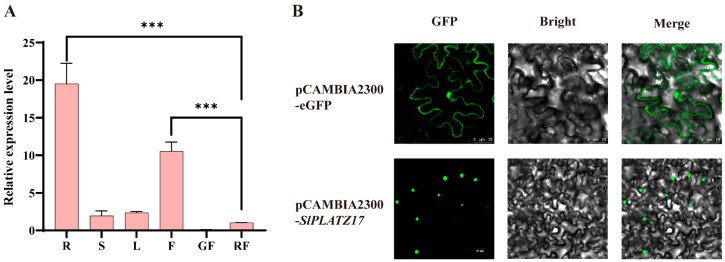
Expression pattern analysis of *SlPLATZ17* in tomato and its subcellular localization. (**A**) Expression of *SlPLATZ17* in R, roots; S, stems; L, leaves; F, flowers; GF, fruit at mature green stage; RF, fruit at mature red stage in WT (***; *p* ≤ 0.001). (**B**) Subcellular localization of SlPLATZ17 protein in tobacco leaves. Bars: 25 µm; 60 µm.

To elucidate the potential function of *SlPLATZ17*, we first analyzed the expression of *SlPLATZ17* in the roots, stems, leaves, flowers, green fruit, and red fruit of AC. The qRT-PCR results revealed that the expression of *SlPLATZ17* was extremely high in flowers and roots, 10–20 times higher than that in red fruit, whereas its expression was relatively low in leaves, stems, and green fruit (Figure 1A). To determine the subcellular localization of the SlPLATZ17 protein, the pCAMBIA2300-*SlPLATZ17* construct was generated and introduced into tobacco leaves. As shown in Figure 1B, compared with that of the pCAMBIA2300-eGFP control, the fluorescence signal of pCAMBIA2300-*SlPLATZ17* was detected only in the nuclei of the cells, indicating that *SlPLATZ17* was localized in the nucleus.

### 2.2. Construction of SlPLATZ17 Overexpression and Knockout Lines

**Figure 2:**
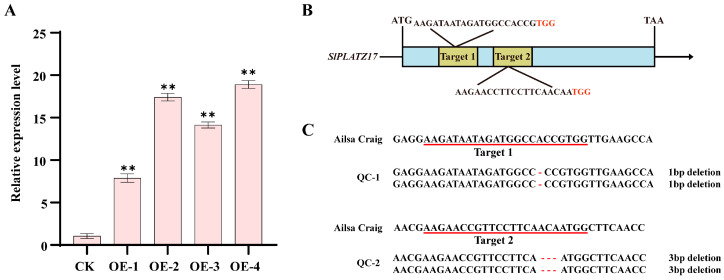
Identification of *SlPLATZ17*-overexpressing and *SlPLATZ17* knockout plants. (**A**) *SlPLATZ17* gene expression in CK and *SlPLATZ17*-overexpressing plants. Data are presented as means ± SEs *(n* = 3). ** (*p* ≤ 0.01) indicates significant differences compared with CK (AC), as determined by one-way ANOVA. (**B**) Schematic diagram of tomato genome editing mediated by CRISPR/Cas9. Arrow indicates direction of genome, from 5′ to 3′. Yellow boxes represent sgRNA target sites, sequences of which are also shown. PAM motifs are marked in red. (**C**) Sequence near mutation site of *SlPLATZ17* gene. Lower part of sgRNA target site is marked with red line.

In this study, we used *SlPLATZ17*-overexpressing T2 generation strains obtained in a previous study and performed fluorescence quantitative PCR for *SlPLATZ17*. Compared with that in the control AC plants, gene expression in all four strains was significantly upregulated, and the OE-2 and OE-4 strains are chosen, which presented greater expression, for the subsequent experiments (Figure 2A). The successful *pYLCRISPR/Cas9-SlPLATZ17* vector was subsequently transformed into the tomato variety AC via Agrobacterium, and four knockout plants were obtained. The designed targets are shown in Figure 2B. The *SlPLATZ17* gene-edited plants from four T0 generation plants were identified via PCR to amplify a 342 bp fragment, and the products were subsequently sent to a sequencing company. Alignment of the sequencing results with the target fragments was performed via DNAMAN software (6.0), and target resolution was performed via the web-based tool DSDecodeM [22]. Through two consecutive generations of planting and testing, we generated two T2 generation pure knockout strains, QC-1 and QC-2 (Figure 2C), which were used for subsequent stress and other experiments.

### 2.3. SlPLATZ17 Overexpression Improves Drought Tolerance in Tomatoes

**Figure 3:**
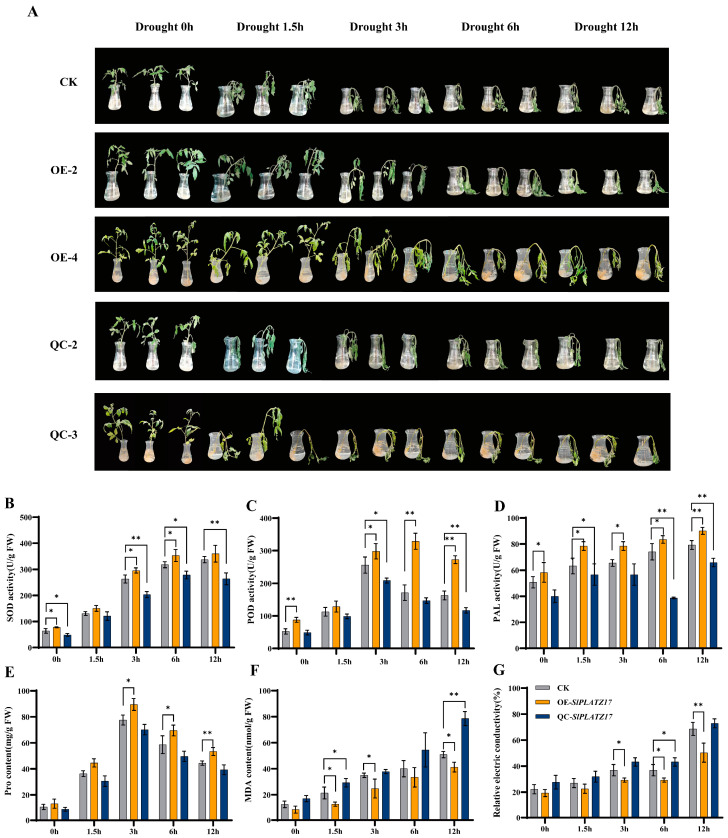
Overexpression of *SlPLATZ17* improved drought tolerance in tomato. (**A**) Changes in phenotypes of different plant lines under drought stress. Analysis of physiological indicators of different plant lines under drought stress: (**B**) SOD activity; (**C**) POD activity; (**D**) PAL activity; (**E**) Pro content; (**F**) MDA content; (**G**) relative electrical conductivity. All treatments were performed in triplicate with biological replicates. * (*p* ≤ 0.05) and ** (*p* ≤ 0.01) indicate significant differences compared with CK at each time point, as analyzed by one-way ANOVA.

Three groups of tomato seedlings were subjected to 15% PEG6000 treatment. As shown in Figure 3A, the leaf blades of the three groups of plants started to wilt, and the stems became bent and drooped at 1.5 h. The degree of stem bending and leaf wilting were most severe in the knockout plants, whereas the overexpression plants performed the best, exhibiting only slightly bent stems; at 3 h, the three groups of plants further wilted, but the overexpression plants performed slightly better, and the remaining two groups performed similarly. The knockout plants presented the most severe degree of wilting, whereas the overexpression plants presented the best performance, with only slightly bent stems. At 6 h, the three groups of plants exhibited further wilting, but the overexpression plants presented slightly better performance, and the remaining two groups presented similar performance. In addition to performing phenotypic observations, we measured the related physiological and biochemical indices, and the results revealed that the four indices, Superoxide dismutase (SOD), Phenylalanine ammonia lyase (PAL), and Peroxidase (POD) activities and proline (Pro) content, were elevated in the overexpression plants but reduced in the knockout plants compared with the wild-type plants overall, and the decrease was significant at some time points (Figure 3B–E). Taken together, these findings indicate that drought-related physiological indices were increased in the overexpression plants but reduced in the knockout plants overall. Measurement of the malondialdehyde (MDA) content and relative conductivity in the three groups of plants revealed that both indicators tended to increase, and the increase was most rapid in the knockout plants, peaking at 12 h. The increase in the overexpression plants was slower than that in the wild-type, with smaller values overall (Figure 3F,G). Overall, the analysis revealed that the cell membranes of the overexpression plants presented the least damage under drought stress, whereas those of the knockout plants presented the most severe damage under drought stress.

**Figure 4:**
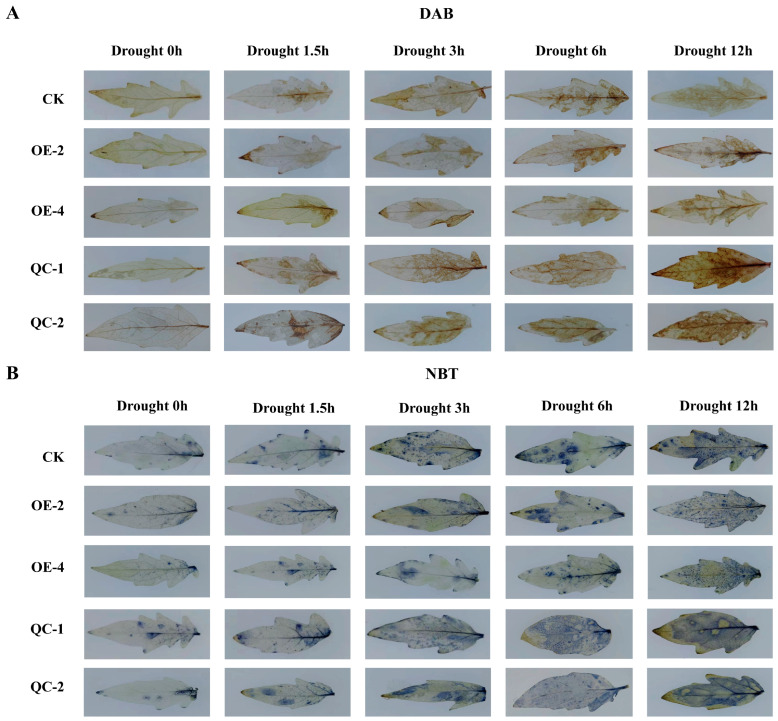
Reactive oxygen species staining in different plant lines under drought stress. (**A**) DAB staining of different lines under drought treatment, with brown representing H_2_O_2_. (**B**) NBT staining of different lines under drought treatment, with blue representing O^2−^.

To further characterize the damage to tomato leaves under stress conditions, we subsequently quantified the ROS content. As shown in Figure 4, with prolonged drought stress, the staining area of the plant leaves gradually expanded, and the colour deepened; however, the degree of staining of the leaves from the overexpression plants was clearly the lightest, and all three groups of leaves were almost completely stained at 12 h. The degree of staining of the knockout plants was the strongest, exhibiting a reddish-brown and dark blue colour, whereas the degree of staining of the overexpression plants was lower than that of the wild-type plants overall, with the smallest staining area and the lightest colour. Under drought stress, *SlPLATZ17* overexpression inhibited the production of ROS in leaves, reduced the damage caused by stress to leaves, and improved the drought tolerance of tomato plants.

### 2.4. SlPLATZ17 Overexpression Improves Salt Tolerance in Tomatoes

**Figure 5:**
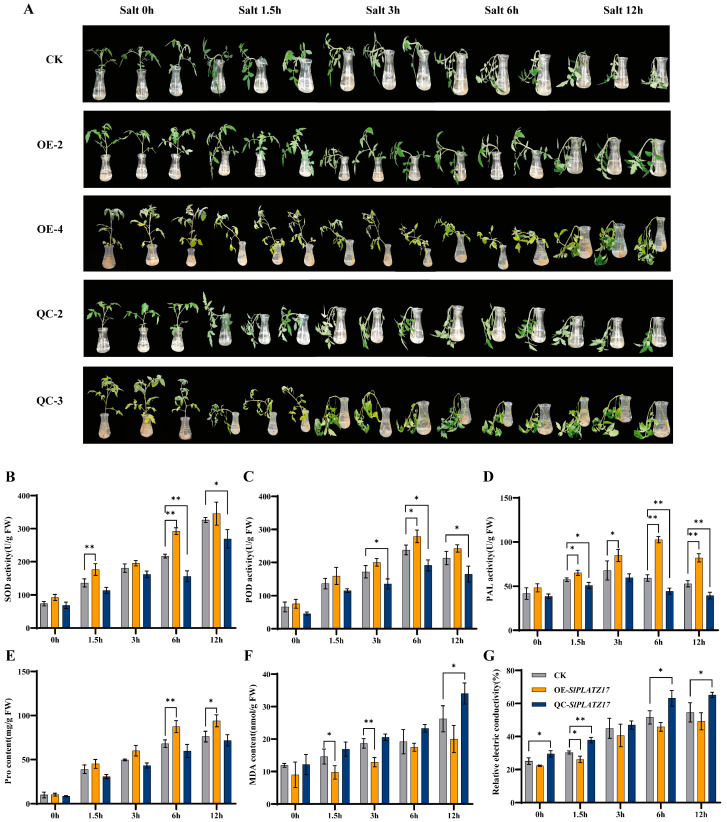
Overexpression of *SlPLATZ17* improved salt tolerance in tomatoes. (**A**) Changes in phenotypes of different plant lines under salt stress. Analysis of physiological indicators of different plant lines under drought stress: (**B**) SOD activity; (**C**) POD activity; (**D**) PAL activity; (**E**) Pro content; (**F**) MDA content; (**G**) relative electrical conductivity. All treatments were performed in triplicate with biological replicates. * (*p* ≤ 0.05) and ** (*p* ≤ 0.01) indicate significant differences compared with CK at each time point, as analyzed by one-way ANOVA.

**Figure 6:**
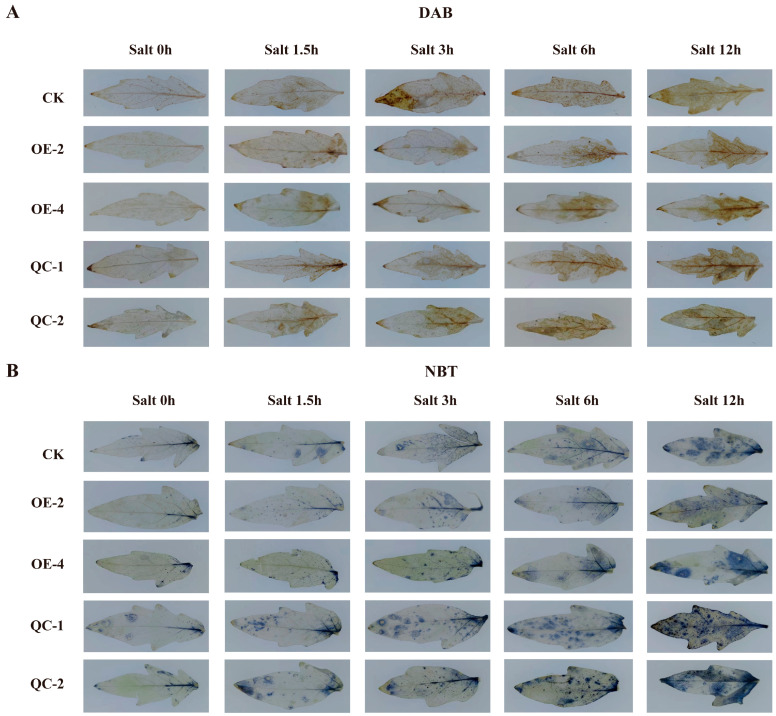
Reactive oxygen species staining in different plant lines under salt stress. (**A**) DAB staining of different lines under salt treatment, with brown representing H_2_O_2_. (**B**) NBT staining of different lines under salt treatment, with blue representing O_2_^−^.

Three groups of tomato seedlings were subjected to 200 mM NaCl salt treatment. As shown in Figure 5A, the stems of all three groups of plants became bent and drooped at 1.5 h of treatment, with the stems of the overexpression plants bending the least and those of the knockout plants bending the most severely; from 3 to 6 h, the stems of the wild-type plants and the knockout plants were completely bent and drooping, whereas the changes in the overexpression plants were not obvious, and the stems of the overexpression plants did not completely droop until 12 h. The stems of the wild-type and knockout plants were completely bent and drooping, whereas the stems of the overexpression plants did not completely droop. The phenotype of the stems of the overexpression plants was not obvious until 12 h, when complete drooping was observed. Moreover, the physiological indices related to the salt resistance of the overexpression plants increased strongly, whereas those of the knockout plants decreased (Figure 5B–E). The MDA content and conductivity of the gene-edited plants were consistently greater than those of the other two groups, indicating that knockdown of the *SlPLATZ17* gene resulted in a high degree of cellular plasma peroxidation, severe damage to the cellular membrane, and severe injury caused by salt stress in the plants (Figure 5F,G). The leaves from the different treatment timepoints were stained; the whole leaves of the knockout plants were reddish-brown or dark blue at 12 h of treatment, whereas those of the overexpression plants showed staining in only the upper part of the leaves, and the degree of staining of the overexpression plants was the lightest among the three groups of plants (Figure 6). Taken together, these results indicate that the overexpression of *SlPLATZ17* reduced the degree of damage caused by salt stress to tomato plants and improves their salt tolerance.

### 2.5. Sequencing Analysis of SlPLATZ17 Transcriptome

We performed transcriptome sequencing analysis for three biological replicates of three groups of *SlPLATZ17* gene-overexpressing plants (OE), knockout plants (QC), and AC plants (CK), with a total of nine samples, and statistical analysis showed that the total number of bases in the individual samples in the filtered data was greater than 6 Gb, the percentage of filtered sequenced bases with a quality level of Q20 or above was greater than 97%, and the average percentage of Q30 bases was greater than 92% (Table S2). In the reference genome comparison analysis, among the nine groups of samples, the number of reads and the proportion of valid reads that were localized to the reference genome were greater than 95%, whereas the number of reads and the proportion of valid reads that were unique compared to the reference genome were greater than 90%, which is a high comparison rate in transcriptome sequencing (Table S3). These results indicated that the transcriptomic data were of high quality and could be subsequently analyzed.

**Figure 7:**
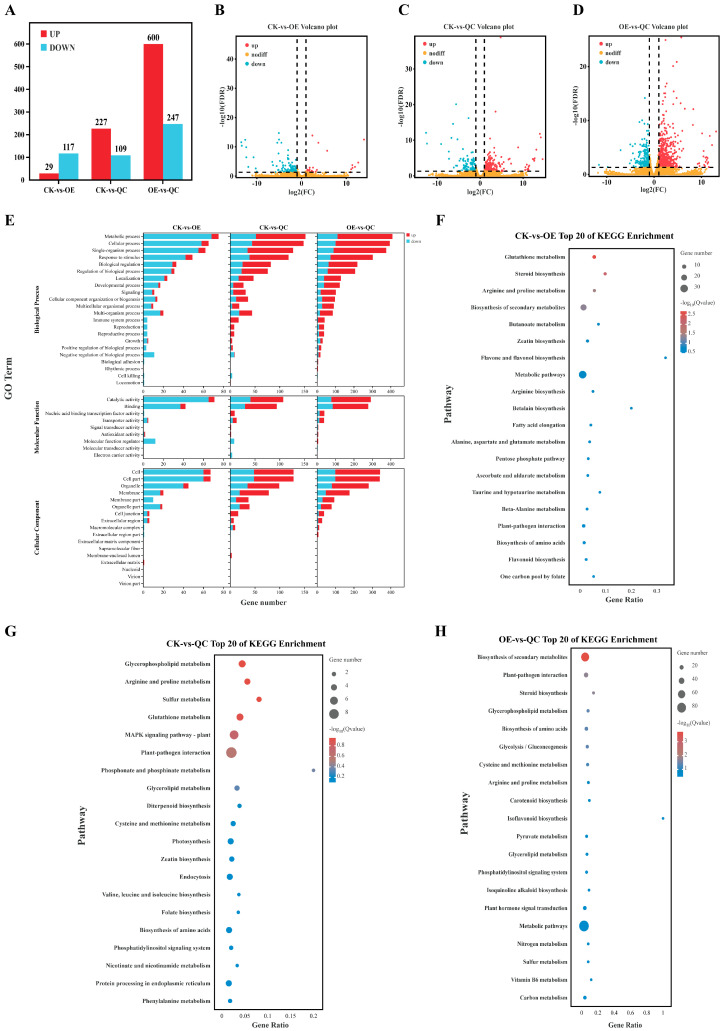
Transcriptome sequencing analysis. (**A**) Statistics of number of differentially expressed genes. (**B**–**D**) Volcano plot of differentially expressed genes between groups. Red, upregulated expression; blue, downregulated expression; orange, no difference in expression. (**E**) GO analysis of differentially expressed genes. (**F**–**H**) Scatter plot of enriched KEGG pathways of differentially expressed genes.

We compared AC plants, OE plants, and gene-edited plants in a pairwise manner on the basis of their gene expression levels in the 0 h transcriptome; compared with OE plants, CK plants presented a lower number of upregulated genes; compared with QC plants, CK plants presented a greater number of upregulated genes (227); and compared with QC plants, OE plants presented 600 upregulated genes and 247 downregulated genes (Figure 7A). On the basis of the significantly differentially expressed genes in the comparison group, we performed volcano plot analysis (Figure 7B). The distribution of upregulated and downregulated is visualized in the figure for a more intuitive depiction. The volcano plot for the OE plants compared with the QC revealed the presence of significantly more upregulated genes and a wider distribution at both ends, indicating a greater degree of genetic differentiation. GO analysis of the three groups of materials, CK, OE, and QC, revealed that the three groups of materials presented the most variation in the number of differentially expressed genes in the biological process category, and in the comparison of two groups of materials, OE and QC, the number of upregulated genes related to biological processes was also the greatest, including genes involved in metabolic processes and cellular processes. The cellular component terms with the greatest number of upregulated genes were the cellular, cellular components, etc.; the molecular function terms with the highest number of upregulated genes were functions such as catalytic activity and nucleic acid binding transcription factor activity (Figure 7C–E). KEGG analysis of the differentially expressed genes was performed on the three groups of materials, and pairwise comparison revealed that the most enriched metabolic pathways in the CK vs. OE group included glutathione metabolism, steroid biosynthesis, arginine and proline metabolism, and secondary metabolism biosynthesis; the differentially abundant metabolites in the CK vs. QC group were enriched mainly in glycerophospholipid metabolism, arginine and proline metabolism, sulphur metabolism, and glutathione metabolism; the pathways with the most significant differences in the OE vs. QC group were secondary metabolism biosynthesis, plant–pathogen interactions, and steroid biosynthesis (Figure 7F).

### 2.6. SlPLATZ17 and POR1 Proteins Have a Reciprocal Relationship

**Figure 8:**
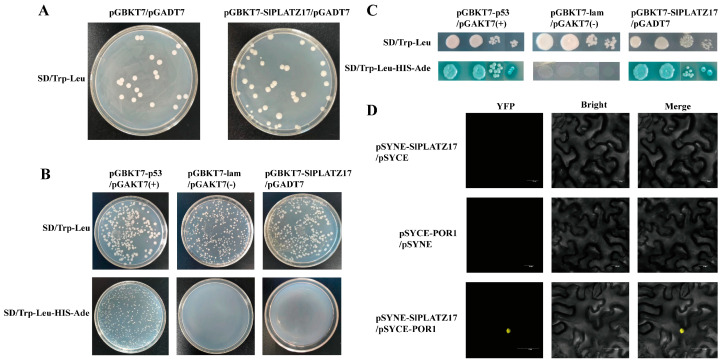
POR1 is an important SlPLATZ17-interacting protein. (**A**) SlPLATZ17 genotoxicity testing. (**B**) *SlPLATZ17* gene autoactivation test. (**C**) Yeast two-hybrid validation of the SlPLATZ17-interacting protein. (**D**) The identification of the SlPLATZ17-interacting proteins via BiFC. Bars: 20 µm.

To screen for proteins that interact with SlPLATZ17, we performed a yeast two-hybrid library screen. First, the toxicity and self-activation of the pGBKT7-SlPLATZ17 bait gene were examined, and it was found that all the transformants grew on SD medium lacking Leu and Trp (SD/Trp-Leu), whereas their growth on SD medium lacking Leu, Trp, His and Ade (SD/Trp-Leu-His-Ade) was effectively inhibited, indicating that *SlPLATZ17* did not have toxic effects and that self-activation did not occur (Figure 8A,B). After screening and analysis, we ultimately selected seven proteins that may have relevant functions for interaction validation (Table S4). Among these, only pGBKT7-SlPLATZ17 and pGADT7-POR1 showed normal growth in SD/Trp-Leu and SD/Trp-Leu-His-Ade + X-α-gal medium, and the clones presented blue colouration (Figure 8C), whereas the clones with the remaining six proteins presented no blue colouration on the quadruple dropout medium. These findings indicated that SlPLATZ17 interacted with the POR1 protein but not with the remaining six proteins. These interactions were further verified via a bimolecular fluorescence complementation (BiFC) assay, which revealed that SlPLATZ17 interacted with POR1 proteins in the nucleus (Figure 8D).

### 2.7. Expression Pattern Analysis and Subcellular Localization of SlPLATZ17-Interacting Protein POR1

**Figure 9:**
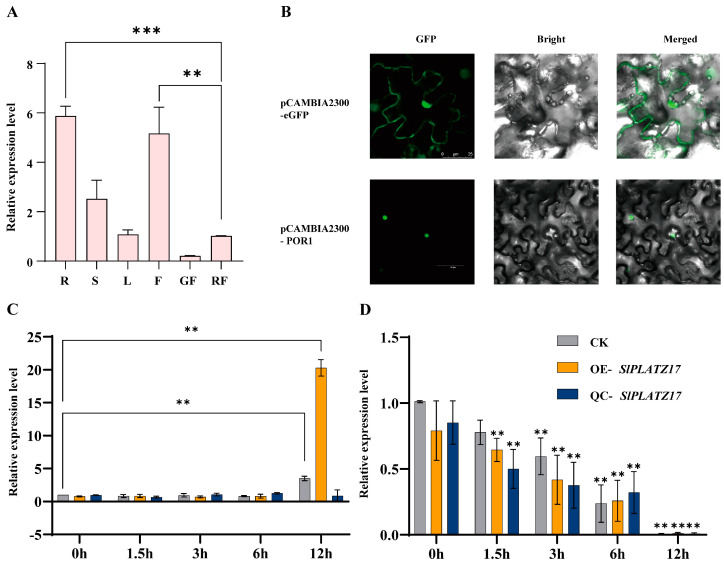
Expression pattern analysis of *POR1* in tomato and its subcellular localization. (**A**) Expression of POR1 in R, roots; S, stems; L, leaves; F, flowers; GF, fruit at mature green stage; RF, fruit at mature red stage in WT. (**B**) Subcellular localization of POR1 protein in tobacco leaves. (**C**) Analysis of expression pattern of POR1 under drought stress. Bars: 25 µm; 60 µm. (**D**) Analysis of expression pattern of POR1 under salt stress. Data are presented as means (±SEs) of three independent biological replicates (**, *p* ≤ 0.01; ***, *p* ≤ 0.001).

We examined the expression of *POR1* in various tissue fractions of tomatoes and found that *POR1* expression was high in the roots and flowers of AC plants, 5- to 8-fold higher than that in red fruit (control) (Figure 9A). Further analysis of its expression pattern under stress conditions revealed that *POR1* expression was low from 0 to 6 h of drought stress, and by 12 h, *POR1* expression increased rapidly in both the AC and OE plants, even exhibiting a 20-fold increase in the OE plants (Figure 9C). Under salt stress, *POR1* expression tended to decrease overall in the three groups of plants, but the change in value was small (Figure 9D). Subcellular localization revealed that POR1 was localized to the nucleus (Figure 9B), which was consistent with the expression profile of *SlPLATZ17*.

### 2.8. Functional Prediction for the SlPLATZ17-Interacting Protein POR1 on the Basis of Transcriptome Sequencing Data

**Figure 10:**
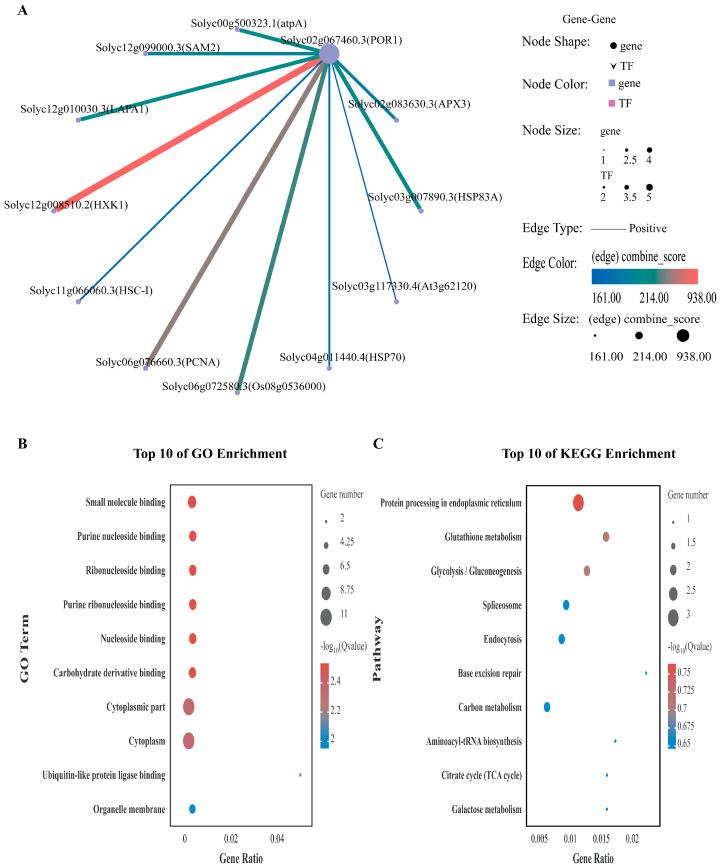
POR1 protein-interacting gene set. (**A**) POR1 protein-interacting gene set. (**B**) GO analysis. (**C**) KEGG analysis.

To further explore the regulatory pathways in which POR1 may be involved, we established a network of interacting genes in the transcriptome database for the protein POR1, as shown in Figure 10A. The set of POR1 protein-interacting genes was subjected to GO and KEGG analyses (Figure 10B,C). The genes that interact with the POR1 protein were enriched mainly in cellular processes, individual tissue processes, metabolic processes, and stimulus responses in the GO analysis, and meticulous delineation revealed them to be involved in small molecule binding, purine-ribonucleoside binding, ribonucleoside binding, purine-ribonucleoside binding, nucleoside binding, carbohydrate derivative binding, cytoplasmic fractions, and the cytoplasm. The genes interacting with POR1 proteins identified via KEGG analyses were enriched in protein processing in the endoplasmic reticulum, glutathione metabolism, glycolysis/gluconeogenesis, and carbon metabolism.

**Figure 11:**
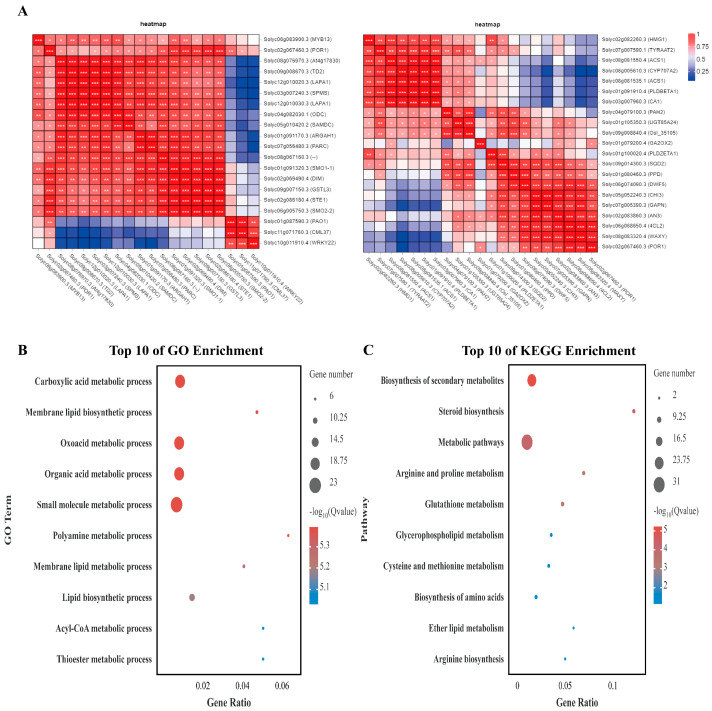
Set of genes coexpressed with POR1 protein. (**A**) Genes coexpressed with POR1 protein. (**B**) GO analysis. (**C**) KEGG analysis (*, *p* ≤ 0.05; **, *p* ≤ 0.01; ***, *p* ≤ 0.001).

We analyzed the correlations of the coexpression of the reciprocal protein POR1 and found that most of the genes were highly correlated with POR1 (Figure 11A). These genes were subsequently analyzed via GO, and most of the genes were enriched in the processes of carboxylic acid metabolism, membrane lipid biosynthesis, oxyacid metabolism, organic acid metabolism, small molecule metabolism, polyamine metabolism, membrane lipid metabolism, and lipid biosynthesis (Figure 11B). KEGG analysis revealed that most of the differentially expressed genes were associated with the biosynthesis of secondary metabolites, steroid biosynthesis, metabolic pathways, arginine and proline metabolism, glutathione metabolism, and other metabolic processes (Figure 11C).

### 2.9. Validation of qRT-PCR Expression Patterns of Important Differentially Expressed Genes

**Figure 12:**
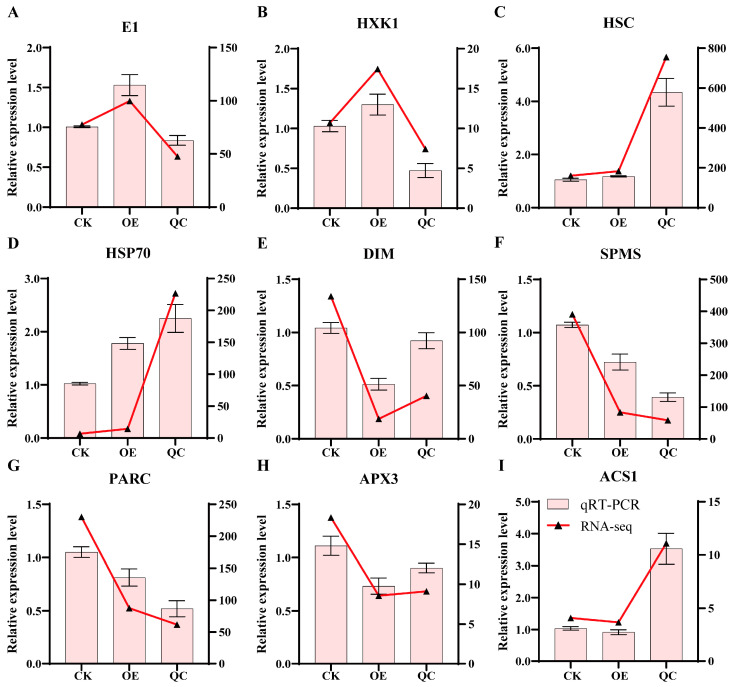
qRT-PCR validation of RNA-Seq results. (**A**,**B**) Relative expression of genes related to glycolysis. (**C**,**D**) Relative expression of genes related to protein processing in endoplasmic reticulum. (**E**) Relative expression of genes related to steroid biosynthesis. (**F**) Relative expression of genes related to arginine and proline metabolism. (**G**,**H**) Relative expression of genes related to glutathione metabolism. (**I**) Relative expression of genes related to secondary metabolism. Vertical coordinate on left represents qRT-PCR results for differentially expressed genes, while vertical coordinate on right represents RNA-seq results. Data are presented as means (±SEs) of three independent biological replicates.

For the important differentially expressed pathways, such as glutathione metabolism and arginine and proline metabolism, screened in the above analysis, we selected nine differentially expressed genes for further expression pattern analysis and validation, as shown in Figure 12. These genes showed obvious expression changes in OE or QC, indicating that the expression of these genes was indeed affected by changes in the expression of the *SlPLATZ17* gene, which may further affect the metabolic pathways in which these genes are located. In addition, the expression patterns in the qRT-PCR results showed the same trend of change as the transcriptome sequencing results, indicating that our RNA-Seq data were accurate and reliable.

## 3. Discussion

### 3.1. SlPLAT17 Can Regulate the Salt and Drought Tolerance of Tomatoes by Reducing the Accumulation of ROS

In the normal growth state of a plant, a relative balance is maintained between the production of ROS and the antioxidant system in its body, which protects the plant from damage [23,24]. However, when a plant is subjected to stress, it is not possible to maintain this balance, and a large amount of ROS accumulates in the plant cells, disrupting the membrane structure of the cells and damaging the corresponding functions [25,26]. In previous research [21], we found that the reduced expression of *SlPLATZ17* decreased the drought and salt tolerance of tomato plants, and we hypothesized that the downregulation of *SlPLATZ17* might lead to the accumulation of free radicals and ROS, thus weakening plant stress tolerance. Here, we used stable overexpression and knockout to further analyze the function of *SlPLATZ17* in stress tolerance and found that the behaviour of the *SlPLATZ17* knockout plants was consistent with that of the gene-silenced plants and that the *SlPLATZ17*-overexpressing plants presented increased drought and salt tolerance, suggesting that the *SlPLATZ17* gene positively regulates the stress tolerance of these plants (Figure 3 and Figure 5). H_2_O_2_ and O_2_^−^ levels can be detected by nitroblue tetrazolium (DAB) and 3,3′-diaminobenzidine (NBT) staining, which allows for the measurement of the amount of ROS produced by plants after stress injury [27]. We stained the leaves of the drought- and salt-treated plants at different times and confirmed that the accumulation of ROS was lowest in the overexpression plants and highest in the knockout plants, which exhibited the most severe damage. In conclusion, these results suggest that *SlPLATZ17* can reduce the production and accumulation of ROS by increasing the free radical scavenging and antioxidant capacity of plants, which in turn modulates the drought and salt tolerance of tomatoes.

### 3.2. SlPLAT17 May Be Involved in the Regulation of Root and Flower Development in Tomato Plants

We screened the *SlPLATZ17* gene in a tomato stress response-related yeast library via yeast two-hybridization and obtained one unreported reciprocal protein in tomato, POR1, after double validation via yeast two-hybrid one-to-one and BiFC assays. An analysis of tissue-specific expression patterns (Figure 1A and Figure 9A) revealed that *SlPLATZ17* and *POR1* presented the highest expression in roots and flowers, whereas the expression changes in other tissues were not obvious, and a previous experimental study revealed that the development of roots and flowers of *SlPLATZ17*-overexpressing plants differed from that of wild-type plants [21]. Therefore, the *SlPLATZ17* gene and POR1 protein may be jointly involved in the regulation of root and flower development in tomato plants. In wheat, the PLATZ transcription factor *GL6* positively regulates grain length by altering cell division and affecting cell proliferation to control the number of cells in the seed grain during spikelet development [28]. In poplar, *PtrPLATZ14* regulates leaf development by affecting the cell size control genes *PtrGRF*/*GIF* and *PtrTCP* [29]. Therefore, POR1 may also play a regulatory role in the expansion or elongation of plant organs together with *SlPLATZ17*, but the specific function of POR1 needs to be investigated via further experiments.

### 3.3. The Expression Pattern of POR1 Under Salt Stress Is Different from That of SlPLATZ17

Both SlPLATZ17 and POR1 were localized in the nucleus (Figure 1B and Figure 9B). BiFC showed that the interaction sites were also located in the nucleus (Figure 8D). The specific nuclear localization of *SlPLATZ17* suggests that it has a specific role as a transcription factor in transcriptional regulation and a synergistic regulatory role with POR1. However, the expression pattern of POR1 under stress was different from that of *SlPLATZ17*, especially under salt treatment, and the expression of *POR1* decreased with increasing treatment time (Figure 9D), whereas the expression of *SlPLATZ17* was significantly greater under stress than under normal conditions. The different individual response periods of the two to stress suggest that the interaction between SlPLATZ17 and POR1 may occur at the later stage of the stress response, and there may be a more complex mode of action between the two, which should be investigated in depth with respect to the specific function of POR1 and the specific mode of regulation with *SlPLATZ17*.

### 3.4. SlPLATZ17 Mainly Exerts Its Functions Through the Glutathione Metabolism and the Arginine and Proline Metabolism Pathways

In further clarifying the mechanism by which the *SlPLATZ17* gene is involved in resistance to abiotic stress in tomato, by transcriptome sequencing, we found that the differentially expressed genes in the *SlPLATZ17*-overexpressing, wild-type, and gene-edited plants were enriched in different metabolic pathways. Kyoto Encyclopedia of Genes and Genomes (KEGG) analysis revealed alterations in several pathways, and the metabolic pathways associated with both the *SlPLATZ17*-overexpressing and the *SlPLATZ17* knockout materials compared with the wild-type plants were related to glutathione metabolism and arginine and proline metabolism (Figure 7). We further established a set of reciprocal genes and genes coexpressed with the reciprocal protein POR1 in the transcriptomic database (Figure 10 and Figure 11) and found that the metabolic pathway involved in plant resistance to stress overlapped with the metabolic pathway via which *SlPLATZ17* functions in glutathione metabolism and arginine and proline metabolism. These results suggest that *SlPLATZ17* most likely functions through the glutathione metabolic pathway as well as the arginine and proline metabolic pathways.

Glutathione (GSH) is a ubiquitous, abundant, and indispensable thiol involved in a variety of biological processes, such as ROS scavenging, redox signalling, sulphur storage and transport, detoxification of hazardous substances, and metabolism of many compounds [30]. The overexpression of the *SbGST* (Tau-like glutathione transferase) gene in transgenic tobacco promotes seed germination and growth under salt stress, and *GST* can be genetically engineered to increase abiotic stress tolerance in plants [31]. The application of exogenous Pro or the transformation of genes involved in Pro metabolism enhances plant tolerance to oxidative stress [2]. Strawberry *FaNAC2* improves tolerance to abiotic stress by regulating proline metabolism [32]. Plant researchers agree that proline accumulation is beneficial to plants, especially after recovery from stress [33]. The maize protein arginine methyltransferase gene plays an important role in flowering regulation and abiotic stress tolerance in Arabidopsis [34]. Therefore, we hypothesized that *SlPLATZ17* is involved in glutathione metabolism pathway when plants are subjected to abiotic stress, which in turn improves the stress tolerance of tomato plants by controlling the accumulation of ROS and other activities involving glutathione and other substances [35]. Simultaneously, the metabolism of arginine and proline maintains the osmotic balance within and outside the cells while scavenging free radicals, thereby enhancing the drought resistance of plants and reducing damage from salt stress. However, in this study, we did not measure the contents of glutathione and arginine and could not determine the specific metabolites through which *SlPLATZ17* and POR1 play regulatory roles; thus, the downstream regulatory mechanisms should be further investigated in detail in subsequent studies.

In this study, we demonstrated the role of the *SlPLATZ17* gene in regulating abiotic stress tolerance in tomatoes by analyzing expression, phenotypic, physiological, and biochemical data. By integrating physiological assessments, molecular techniques, and transcriptomics, we have provided new insights into the biological function of *SlPLAZT17* in the regulation of drought and salt tolerance, and the phenotypes observed in transgenic plants are expected to be used to improve drought and salt tolerance in crops. In future work, we will conduct a more detailed investigation into the downstream regulatory mechanisms of the *SlPLATZ17* gene and hope to widely apply the *SlPLAZT17* gene in the breeding of salt- and drought-tolerant crop varieties.

## 4. Materials and Methods

### 4.1. Generation of SlPLATZ17 Overexpression and Knockout Strains

In this study, tomato *SlPLATZ17*-overexpressing T2 generation lines obtained in our previous study were used [21]. *SlPLATZ17* knockout plants were obtained via CRISPR/Cas9 technology. First, the target design programme on the website http://skl.scau.edu.cn/ (accessed on 15 January 2023) was used to design the target. Then, primers for constructing expression boxes were generated via the “primer Design V” programme (http://skl.scau.edu.cn/primerdesign/vector/, accessed on 15 January 2023) (Table S1). The pYLCRISPR/Cas9-SlPLATZ17 vector was constructed and transformed into the DH10B colonic receptor for the Agrobacterium-mediated genetic transformation of tomatoes. The editing results were verified via PCR and Sanger sequencing, and the T0 knockout-positive seedlings were naturally grown for two generations to obtain the T2 generation with genetically stable double allelic mutations for testing.

### 4.2. Plant Stress Treatments

The T2 generation of *SlPLATZ17*-overexpressing, T2 generation of gene knockout, and wild-type AC (Ailsa Craig) tomato seedlings were placed in a light incubator for cultivation (light period, 16 h; light intensity, 35,000 lx; temperature, 20–25 °C; dark period, 8 h; temperature, 13–15 °C; and relative humidity, 45%). When the seedlings grew to the 4-leaf and 1-heart stage, uniformly growing plants were selected and cultured in Hoagland nutrient solution for 12 h. Afterwards, the plants were divided into two groups and subjected to drought treatment with 15% PEG6000 and salt treatment with 200 mM NaCl. Each treatment was replicated three times, and the phenotypic changes in the plants were observed at 0, 1.5, 3, 6, and 12 h after the treatment. The samples were obtained, snap-frozen in liquid nitrogen, and stored at −80 °C until used for the determination of physiological indices.

### 4.3. Quantitative Real-Time PCR (qRT-PCR) Analysis

Total RNA was extracted from samples using Trizol. cDNA synthesis was performed using the M-MLVRTase Kit (TaKaRa, Dalian, China). qRT-PCR was performed using the iQ5 system. For each qRT-PCR, the mixture consisted of 10 μL SYBR^®^ Green Master Mix, 0.5 µL each of forward primer and reverse primer, 1 µL of diluted cDNA, and 8 µL of ddH_2_O in a 20 µL reaction. The reaction was carried out as follows: 95 °C for 5 min, followed by 40 cycles of 94 °C for 5 s, 60 °C for 15 s, and 72 °C for 10 s. The 2^−∆∆CT^ method was used for data analysis, and *Actin-7* was used as a reference gene. All specific primers used for qRT-PCR are listed in Table S1. Each qRT-PCR assay included three biological replicates.

### 4.4. Subcellular Localization

The coding sequence (CDS) of the target gene without a stop codon was amplified and fused with the *pCAMBIA2300-eGFP* vector to obtain the *pCAMBIA2300*-target gene construct, which was subsequently introduced into *Agrobacterium rhizogenes* GV3101. The recombinant Agrobacterium cells were subsequently transformed into tobacco leaves. After two days of incubation in the dark, fluorescence signals were detected with a laser confocal microscope (LSM800, Zeiss, Jena, Germany). The specific primers used for vector construction are listed in Table S1.

### 4.5. Determination of Physiological Indicators

SOD activity was determined following the methods of Beyer and Fridovich [36], and POD activity was determined according to the methods of Chance and Maehly [37]. The proline content in leaves was estimated via the methods of Claussen [38], and the MDA content was estimated via the methods of Cervilla et al. [39]. ROS were detected using via nitroblue tetrazolium (NBT) and 3,3′-diaminobenzidine (DAB) staining methods [40,41]. The blade conductivity was measured with an FE38-ST conductivity metre.

### 4.6. Yeast Two-Hybrid Screening

To identify *SlPLATZ17*-interacting proteins, we used a yeast two-hybrid library screening system. The CDS of *SlPLATZ17* was inserted into pGBKT7 (BD) to construct the pGBKT7-SlPLATZ17 bait plasmid. In addition, a positive control (pGBKT7-p53/pGADT7-T), a negative control (pGBKT7-lam/pGADT7-T), and an empty plasmid control (pGBKT7/pGADT7-T) were used. First, the bait gene pGBKT7-SlPLATZ17 was tested for toxicity and self-activation. The pGBKT7-SlPLATZ17 plasmid and the two-hybrid library plasmid pGADT7 (AD) were then integrated into the Y2HGold yeast strain via cotransformation. The transformants were screened with selective quadruple dropout SD medium (SD/-Trp-Leu-His-Ade) and X-α-gal. After 3–5 days of incubation at 30 °C, single colonies that turned blue on the medium were subjected to Sanger sequencing to obtain sequences for homology analysis. In addition, candidate interacting proteins were screened via yeast two-hybrid one-to-one validation to identify proteins that interact with SlPLATZ17.

### 4.7. Bimolecular Fluorescence Complementation (BiFC) Experiment

The CDSs of *SlPLATZ17* and *POR1* without a terminator were constructed in the pSYNE-YFP vector and pSYCE-YFP vector, respectively, followed by infiltration in tobacco, and yellow fluorescent signals were captured under a laser confocal microscope.

### 4.8. Transcriptomic Analysis

Young leaves of *SlPLATZ17* gene-overexpressing plants, *SlPLATZ17* knockout plants, and CK (AC) plants at the 4-leaf–1-heart stage were collected in three physiological replicates, with a total of nine samples, and stored at −80 °C after quick freezing in liquid nitrogen. After the RNA was extracted, it was sent to Gene Denovo Biotechnology for subsequent library construction and sequencing. To ensure data quality, we removed various types of low-quality reads and then used HISAT2 for alignment analysis on the basis of the reference genome [42]. On the basis of the results of the HISAT2 comparison, the transcripts were then reconstructed via Stringtie, the expression of all the genes in each sample was calculated via RSEM, and GO analysis, KEGG analysis, etc., were performed on the differentially expressed genes (DEGs). We then established the protein POR1 interacting gene set and co-expressed gene set to find metabolic pathways.

### 4.9. Statistical Analysis

Univariate analysis of variance (ANOVA) was performed on the data using SPSS (26.0) software. Post hoc multiple comparisons were conducted using the least significant difference (LSD) and Duncan’s tests. Asterisks (*, **, and ***) are used to denote significant differences (*p* ≤ 0.05, *p* ≤ 0.01, and *p* ≤ 0.001, respectively). The data in this report were obtained from a minimum of three independent experiments, each comprising three technical replicates.

## 5. Conclusions

We report that the PLATZ transcription factor *SlPLATZ17* is involved in regulating tomato resistance to drought and salt stress. The overexpression of *SlPLATZ17* increased seedling resistance to PEG6000 and NaCl, whereas knockout of the *SlPLATZ17* gene exacerbated the damage caused by these stressors to the plants. Furthermore, our study revealed that SlPLATZ17 interacts with POR1 in response to stress and may regulate the accumulation of ROS through pathways involving glutathione metabolism and arginine and proline metabolism. In summary, this research identified the role of *SlPLATZ17* in the response to salt and drought stress, expanding our understanding of the biological functions of PLATZ transcription factors in plants and providing new targets for future studies aimed at enhancing plant resistance.

## Data Availability

All data are displayed in the manuscript and Supplementary Files.

## Supplementary Materials

The following supporting information can be downloaded at: https://www.mdpi.com/article/10.3390/ijms26051836/s1.
