# Supplementary material for: The Overexpression of SlPLATZ17 Can Increase the Tolerance of Tomatoes to Drought and Salt Stress
Source: Int J Mol Sci. 2025 Feb 21;26(5):1836. doi: 10.3390/ijms26051836 (PMC11898940; doi:10.3390/ijms26051836)
Supplement: Supplementary file 1 [file ijms-26-01836-s001.zip › ijms-3435443-supplementary.pdf]

Supplementary Materials

Table S1. All the primer sequences used in this study

**Primer name**	**Forward primer Sequence (5'→3')**	**Reverse primer Sequence (5'→3')**
**For gene editing CRISPR/Cas9 target primers**		
SlPLATZ17 gRT1	AGATAATAGATGGCCACCGGTTTTAGAGCTAGAAAT	CGGTGGCCATCTATTATCTTGACCAATGTTGCTCC
SlPLATZ17 gRT2	AGAACCGTTCCTTCAACAAGTTTTAGAGCTAGAAAT	TTGTTGAAGGAACGGTTCTTGACCAATGGTGCTTTG
**For overexpression and knockout plant validation**		
OE-*SlPLATZ17*	AAGGAAGTTCATTTCATTTGGAGAG	TCCAGCTCGACCAGGATGGG
QC-SlPLATZ17	GATCGATAATCAAGACACCACTG	CTGAATGGCACGATGGTCC
**For express pattern analysis**		
Actin-7	CCTCAGCACATTCCAGCAG	CCACCAAACTTCTCCATCCC
SlPLATZ17	CAGCACAAGCAACAAGGTCC	TCTTCGCTTGGCTGTTCTGT
POR1	TGTTGGGGCTGAGGTGAATC	TCCTTGCCTTCACACTGGTC
**For subcellular localization**		
pCAMBIA2300-*SlPLATZ17*	CGGGGGACGAGCTCGGTACCATGGGAGCTGGAGGACCTGATG	GGTGTCGACTCTAGAGGATCCATAACCTATAACTAGTCCTCCCATTGG
pCAMBIA2300-*POR1*	CGGGGGACGAGCTCGGTACCATGGGGAAGGGTCCTGG	GGTGTCGACTCTAGAGGATCCTGGCTTGAGAGCCAAAGC
**For yeast two-hybrid**		
pGBKT7-*SlPLATZ17*	ATGGCCATGGAGGCCGAATTCATGGGAGCTGGAGGACCTGATG	CCGCTGCAGGTCGACGGATCCTTAATAACCTATAACTAGTCCTC
POR1	GCCATGGAGGCCAGTGAATTCATGGGGAAGGGTCCTGGACT	CAGCTCGAGCTCGATGGATCCCTATGGCTTGAGAGCCAAAG
L15	GCCATGGAGGCCAGTGAATTCATGGGTGCTTACACTTATGTGTC	CAGCTCGAGCTCGATGGATCCTCAACGGTAACGACGGAGA
G6P1E	GCCATGGAGGCCAGTGAATTCATGAAGTTGAAGTTGGTAGC	CAGCTCGAGCTCGATGGATCCTTAGCCATAAATAACTTTCCTAG
Dr1	GCCATGGAGGCCAGTGAATTCATGGAACCTATGGATATCGTT	CAGCTCGAGCTCGATGGATCCTTAGCTATTCAAGGTTTGCTCTGC
PETE	GCCATGGAGGCCAGTGAATTCATGGCCACTGTTACCTCTGCT	CAGCTCGAGCTCGATGGATCCTTAGTTGACAGTAACTTTGCC
CAB13	GCCATGGAGGCCAGTGAATTCATGGCATCAATGGCAGC	CAGCTCGAGCTCGATGGATCCCTAAGCTCCAGGAACAAACTTAG
E1	GCCATGGAGGCCAGTGAATTCATGGCTGCATCAGCCTC	CAGCTCGAGCTCGATGGATCCCATGACATGAGCAGTGCC
AD	TAATACGACTCACTATAGGGCGAGC	AGATGGTGCACGATGCACAG
**For interacting protein BiFC validation**		
pSYNE-SlPLATZ17	TGGCGCGCCACTAGTGGATCCATGGGAGCTGGAGGACCTG	CTCCATCCCGGGAGCGGTACCATAACCTATAACTAGTCCTCCCA
pSYCE-POR1	TGGCGCGCCACTAGTGGATCCATGGGGAAGGGTCCTGG	GTACATCCCGGGAGCGGTACCTGGCTTGAGAGCCAAAGC
**For validation of qRT-PCR expression patterns of important differentially expressed genes**		
E1	GGCGCTGAAATCTGTGCATC	CTGCACGAACGATGTCCTCT
HXK1	AACCTTCCTACTGGGGATGAA	CGCTTTTCTTTCCCACCCAA
APX3	TGTCCCTGGTAGAACGGAATC	GTGGCCAGTTTGAGACCTCC
HSC	CCAAGGGAACAGGACGACTC	GACGCTTGGCATCAAACACG
HSP70	ACCAACACTGTTTTCGACGC	GTCCCGGGATGACCTTGAAA
DIM	TCATCGCGAGATGGAGGTAT	TGTCACCCTGCCTTTTGTGT
SPMS	TTGCAAAGATAGCCAGGGCA	ACCCAATAACGCCACTAGGA
PARC	TGGCAAACCAATTTGCGAGT	TCCTCCCAGAATCATACAACTTC
ACS1	TGCCAGAGTTTAGACAAGCAGT	GGTGTTGGGACCAAAAAGGC

Table S2. Summary of Illumina transcriptome assembly

Sample name	Raw Data （bp）	Clean Data （bp）	Total Clean Bases (Gb)	Clean ReadsQ20(%)	Clean ReadsQ30(%)	GC content (%)
CK-1	48743938	48598538	7.24	97.40	92.55	43.08
CK-2	43855614	43733006	6.52	97.89	93.83	42.91
CK-3	43796628	43671170	6.50	97.81	93.72	43.09
OE-1	42506268	42400874	6.31	98.00	94.06	42.87
OE-2	43539234	43420824	6.46	97.82	93.71	42.87
OE-3	44838948	44706158	6.66	97.98	94.10	42.82
QC-1	44963744	44841348	6.68	97.77	93.55	42.71
QC-2	44914928	44797366	6.67	97.92	93.91	43.10
QC-3	46549372	46432956	6.91	97.66	93.33	42.92

Note: Raw Data represents the number of raw reads, and Clean Data represents the number of high -quality reads.

Table S3. Statistics comparison with reference genome

Sample name	Total Clean Reads (M)	Total Mapped (%)	Uniquely Mapping (%)
CK-1	48.33	46253223 (95.70%)	43953752 (90.94%)
CK-2	43.52	41878051 (96.23%)	40272491 (92.54%)
CK-3	43.48	41733016 (95.98%)	39820840 (91.58%)
OE-1	42.22	40832914 (96.72%)	39209821 (92.87%)
OE-2	43.28	41669052 (96.28%)	40186133 (92.86%)
OE-3	44.57	42837137 (96.11%)	41349768 (92.78%)
QC-1	44.71	43032251 (96.23%)	41666910 (93.18%)
QC-2	44.66	43024634 (96.34%)	41190155 (92.23%)
QC-3	46.27	44470100 (96.11%)	42811583 (92.52%)

Table S4. SlPLATZ17 interaction protein validation list

NCBI database ID	Gene Name	Acronyms	CDS lengths (bp)
101264394	Solanum lycopersicum mitochondrial outer membrane protein porin of 34 kDa	POR1	830
101252296	60S ribosomal protein L15	L15	614
101255458	putative glucose-6-phosphate 1-epimerase	G6P1E	1020
101265446	protein Dr1 homolog	Dr1	476
544053	PETE plastocyanin, chloroplastic	PETE	513
101243766	CAB13 chlorophyll a-b binding protein 13, chloroplastic	CAB13	798
101256654	pyruvate dehydrogenase E1 component subunit alpha-3, chloroplastic	E1	1322
